# The Role of Epidemiology in Challenging the HIV/AIDS Pandemic

**DOI:** 10.2188/jea.11.95

**Published:** 2007-11-30

**Authors:** Roger Detels

**Affiliations:** University of California, Los Angeles School of Public Health, Los Angeles, California, USA.

**Keywords:** HIV/AIDS, intervention, HAART, cohort, pandemic, surveillance

## Abstract

The HIV/AIDS pandemic has challenged the resourcefulness of epidemiology and epidemiologists. In response to the challenge, epidemiologists have used existing epidemiologic strategies, expanded existing strategies, and developed new strategies to answer key questions about the transmission of HIV, the natural history of HIV at the molecular, host, and community levels, for evaluation of treatment effectiveness and intervention strategies, and to inform public health policy. In responding to the challenge of the pandemic, epidemiologists have also increasingly collaborated with scientists from other disciplines, particularly immunology, virology, and the behavioral sciences. Examples of the application of these epidemiologic strategies are presented.

## BACKGROUND

For no other disease has epidemiology played such an important role in describing the key characteristics as for the human immunodeficiency virus (HIV) and its resulting disease, acquired immunodeficiency syndrome (AIDS). These characteristics have included the spread of the agent, the biologic mechanisms involved in the establishment of infection by the agent, the mechanisms used by the body to defend itself against the agent, the psychosocial impact of the epidemic, and the evaluation of strategies for intervention in the disease process and prevention of the spread of the disease in the community. This information has been used to formulate public health policy. In less than twenty years scientists have come to know more about this disease than for most other diseases about which we have known for years. Epidemiology has been a major strategy to elucidate that knowledge.

In this paper I will describe and give examples of the specific strategies used to challenge the HIV/AIDS epidemic. Implicit in this discussion is that epidemiology is most powerful when it is used in conjunction with other scientific disciplines such as immunology, virology, genetics, and the psychosocial sciences. Thus, many of the examples which I will present are actually the result of collaboration of epidemiologists with scientists from these other disciplines. I will also briefly present an example of a collaborative cohort study among men who have sex with men which has been a template for similar studies among other risk groups.

## MONITORING THE EPIDEMIC

**Figure 1.**
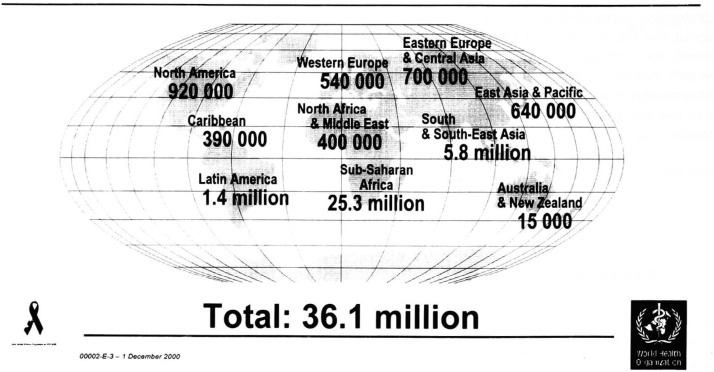
Adults and children estimated to be living with HIV/AIDS as of end 2000.

The estimated distribution of HIV in the various regions of the world, compiled by the Joint United Nations Programme on HIV/AIDS (UNAIDS) and the World Health Organization (WHO), is shown in Figure 1^1^^)^. These estimates have been derived from the reported prevalence of AIDS, surveys for HIV prevalence (most often in risk groups), and knowledge of the degree of under reporting of HIV infections and AIDS cases. From these estimates it is clear that the major focus of the epidemic is currently in sub-Saharan Africa, but that South and Southeast Asia are also major foci of the epidemic. From these estimates it is also clear that more than 95% of new HIV infections are occurring in developing countries, which are least able to cope with the epidemic and that over 50% of infections are occurring in 15-24 year olds. This last statistic is particularly tragic in that this age group represents the future of any country. In some countries of sub-Saharan Africa, HIV/AIDS prevalence rates in sexually active adults have exceeded 30%.

Descriptive epidemiologic studies in various countries of the world have also demonstrated that the pandemic is actually many epidemics, each with its own characteristics. Thus, in the countries of the developed world the major affected groups are men who have sex with men and injecting drug users, whereas in the developing countries of the world, primarily in sub-Saharan Africa, the largest risk group is heterosexuals. In the countries of Southeast Asia the initial group to become infected has been injecting drug users, but the epidemic has spread rapidly from them to other risk groups, and through them to the general heterosexual population.

**Figure 2.**
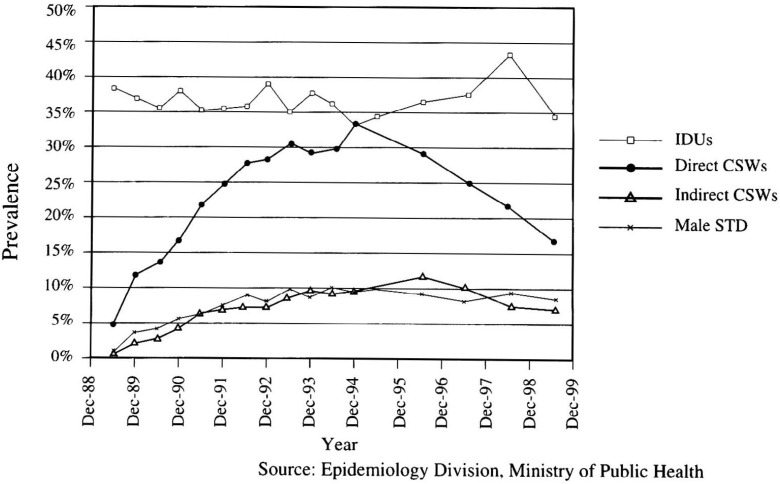
HIV prevalence from national sentinel surveillance among high-risk groups in Thailand, June 1989 - June 1999.

Sentinel surveillance has been a particularly useful epidemiologic strategy for monitoring the epidemic. Perhaps the most effective sentinel surveillance program has been that mounted by Thailand in 1989. Figure 2 presents the results of sentinel surveillance among injecting drug users, “direct” brothel-based commercial sex workers, “indirect” escort-type sex workers, and males attending sexually transmitted disease (STD) clinics from 1989 through June 1999. It is clear that by the time the sentinel surveillance program had begun, the HIV epidemic was already well established among injecting drug users, but soon spread primarily to direct brothel-based sex workers. The results of the sentinel surveillance were used to inform health policy decisions regarding the appropriate intervention strategies to implement such as the “100% condom campaign” and targeting brothels and military recruits, in particular, for intensive intervention. It is clear that the intervention strategies implemented by the Thais, on the basis of surveillance results, had a significant impact on reducing the prevalence of HIV among direct brothel-based sex workers, but had little, if any, impact on the epidemic among injecting drug users. Thus, sentinel surveillance has been useful for documenting the reservoirs of HIV infection, the spread of HIV infection among risk groups, and the impact of intervention strategies. In Thailand, sentinel surveillance has made it clear that while the intervention strategies directed at heterosexual transmission have had some success, there is still a need to implement effective intervention strategies among injecting drug users.

## THE MULTICENTER AIDS COHORT STUDY (MACS)

An example of an epidemiologic cohort study which has generated considerable information about many aspects of HIV disease progression has been the Multicenter AIDS Cohort Study^2^^)^. This study among men who have sex with men, which originated in 1984 and continues today, has been the template for additional studies in other risk groups both in the United States and elsewhere. The men in the MACS provide a detailed history of sexual activities, illnesses and medications, undergo a short physical examination, and donate blood specimens every three to six months for both immediate testing and for repositories which are maintained for future studies which become possible because of the development of new technologies. At the time of the initiation of the MACS, the HIV status of the participants was unknown because the serologic test for HIV had not yet been developed. Thus, the HIV status of the participants had to be determined retrospectively using specimens in the repository.

The MACS has documented the natural history of HIV infection from time of infection (seroconversion) to development of AIDS and death. It has documented four major groups of interest: 1) persistently seronegative men who have had many exposures to HIV, 2) HIV-infected individuals with rapid progression to AIDS and death, 3) HIV-infected individuals who maintain their immune capacity over many years, and 4) individuals who survive for several years despite having low levels of key immune cells. The persistently seronegative men and those who survive for more than a decade with a relatively intact immune system have provided and will continue to provide vital information about strategies used by the body to defend itself successfully against HIV. An understanding of these biologic strategies will play a key role in development of an effective vaccine and therapeutic strategies. The MACS has also contributed papers on behavioral risk factors for infection and progression of HIV disease, molecular factors involved in susceptibility to HIV infection and progression of HIV disease, and the effectiveness of different therapeutic regimens.

**Figure 3.**
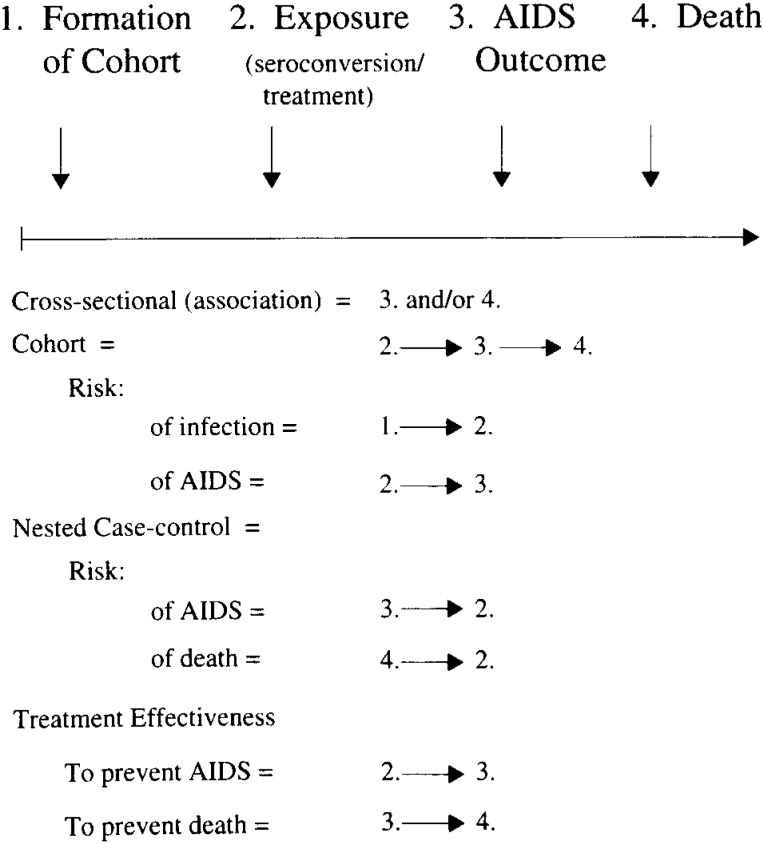
Study design for multicenter AIDS cohort study.

Figure 3 provides examples of the strategies developed and used by the MACS in the study of HIV/AIDS. Among individuals who were not infected with HIV but became infected while under observation, we have documented their status at entry into the MACS cohort, their seroconversion, and the development of AIDS and death in those who progress. The natural history is determined by observation of the progression of the men in the cohort from exposure to HIV to death. Factors associated with infection or development of AIDS can be determined by cross-sectional studies which document the higher prevalence of specific factors among those with HIV infection or AIDS. Risk of infection can be determined by observation of the factors associated with transition from uninfected to infected, risk of AIDS by observation of the factors associated with transition from infection to AIDS, and risk of death by observation of the factors associated with the transition from AIDS to death. In addition, from a cohort study, a “nested case-control” strategy can be used to identify those who have become infected, developed AIDS, or died, looking retrospectively to determine what factors were association with progression. This strategy is particularly effective when new technologies elucidating biologic mechanisms become available which were not available at the time the transition occurred. The efficacy of treatment to prevent development of AIDS can be determined by observing the progression from seroconversion (infection) of individuals with and without treatment to AIDS or from AIDS to death.

## THE NATURAL HISTORY OF HIV INFECTION

Epidemiologic studies have established the modes of HIV transmission. Cross-sectional studies have demonstrated an association between HIV infection and specific activities, including anal intercourse, sharing of drug-injecting equipment, vaginal intercourse, birth from an infected mother, and exposure to infected blood. These associations have been confirmed as risk activities by cohort studies establishing the risk of HIV infection associated with each of these activities. Table 1, taken from the MACS cohort study of men who have sex with men, demonstrates the risk of HIV infection associated with insertive and receptive anal intercourse^3^^)^. From these epidemiologic studies the major risk groups for HIV infection have been identified. Epidemiologic studies have also confirmed that the risk groups of importance in transmission of HIV differ between different communities.

**Table 1.** Risk ratio for HIV-1 seroconversion over 2 years stratified by reported anal-genital intercourse in the multicenter AIDS cohort study.

Reported anal-genital intercourse in previous 12 months	Number seroconverting	Estimated incidence*	Risk Ratio
			
Both receptive and insertive			
	191	.038	76

Receptive, no insertive			
	14	.018	36

Insertive, no receptive			
	10	.005	10

No anal-genital	1	.0005	1

* per 6 month person-interval

**Figure 4.**
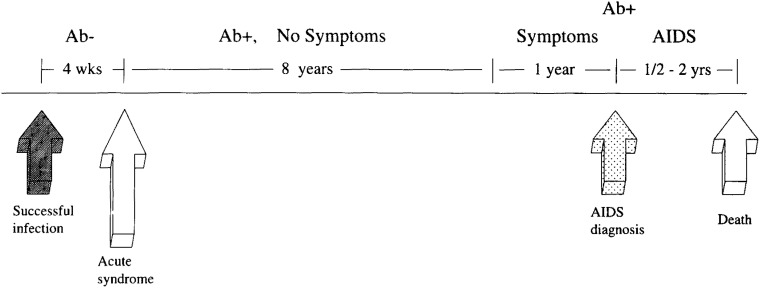
Natural history of untreated HIV infection.

Cohort studies have established the natural history of infection in the absence of treatment at both the individual level and at the community level. Figure 4 presents a time line which gives the major events in the progression from untreated HIV infection to AIDS and death and the average intervals between these major events. Although the average time between successful infection and development of AIDS is nine years, these cohort studies have also demonstrated that progression from exposure to AIDS can occur within one year in some individuals, and in more than a decade in other individuals^4^^)^.

With an understanding of the natural history of HIV infection in the individual, it has been possible to develop epidemiologic models of the natural history of HIV infection in the community. Table 2 presents a model of the spread of HIV infection in a community based on the experience in Thailand. Using the assumption that the interval from HIV infection to AIDS is exactly ten years, the first case of AIDS does not appear until year ten. By then there are already 15,000 HIV infections. The first death from AIDS does not occur until year 12, by which time the number of HIV infections is almost 44,000. Although this model assumes exact intervals between successful infection and AIDS and between AIDS and death, it is, nonetheless, useful in demonstrating to health officials that by the time they are seeing their first cases of AIDS in their community the epidemic is already well established and intervention strategies need to be implemented.

**Table 2.** Incidence, prevalence and mortality of HIV/AIDS (example).

	HIV Infected Persons		AIDS Cases		AIDS Deaths
	
Year	Incident	Prevalent	Incident	Prevalent	
1	1	1	0	0	0
2	6	7	0	0	0
3	15	22	0	0	0
4	48	70	0	0	0
5	195	265	0	0	0
6	359	624	0	0	0
7	702	1,326	0	0	0
8	1,512	2,838	0	0	0
9	4,068	6,906	0	0	0
10	8,131	15,036	1	1	0
11	12,317	27,347	6	7	0
12	16,520	43,852	15	21	1
13	21,649	65,453	48	63	6
14	26,157	91,415	195	243	15
15	29,857	120,913	359	554	48

Clinical epidemiologic studies have also demonstrated the outcomes of exposure to HIV. These include unsuccessful infection and successful infection. Successfully infected individuals may proceed rapidly or slowly to AIDS, which may be manifested as dementia, wasting disease, an opportunistic infection, or an opportunistic malignancy. Two groups revealed by these cohort studies which are of particular interest are those individuals who are repeatedly exposed to HIV but do not become infected and infected individuals who maintain a functional immune capacity for a decade or more^5^^-^^7^^)^. The first group can provide information about natural host factors in some individuals which allow them to resist HIV infection. These factors may then be adapted for administration to individuals without these natural host factors making them resistant to HIV infection. The second group can provide information about the ability of some individuals to contain the virus, preventing the deterioration of the immune system and the development of AIDS. Knowledge of these factors can help to develop drugs and/or strategies to prevent progression to AIDS in individuals who lack this natural ability.

## ELUCIDATION OF MOLECULAR MECHANISMS IN HIV DISEASE

**Figure 5.**
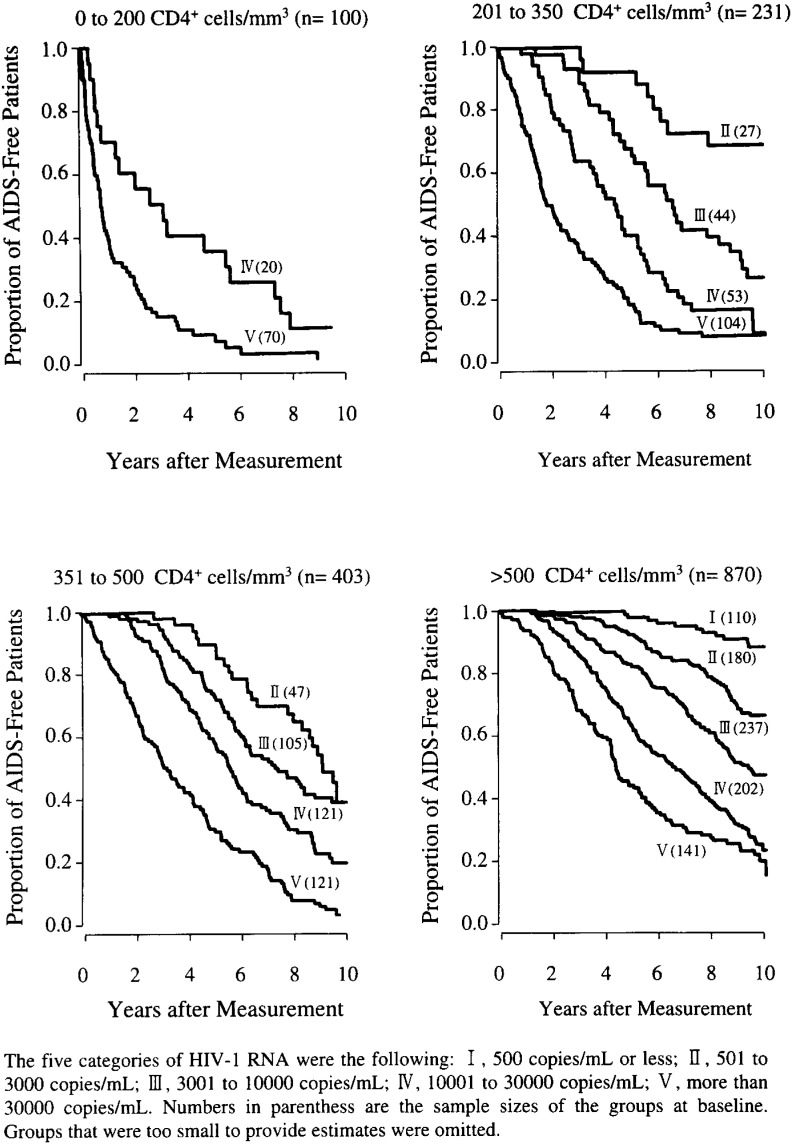
Kaplan-meier curves showing acquired immunodeficiency syndrome (AIDS)-free survival by human immunodeficiency virus type 1 (HIV-1) RNA category among groups with different baseline CD4^+^ lymphocyte counts.

Epidemiologic studies are also useful in demonstrating immunologic changes associated with progression of disease. For example, an early cross-sectional study done before identification of the causative agent of AIDS demonstrated that the practice of receptive anal intercourse was associated with an increase in a particular subset of immune cells, the CD8^+^ cells^8^^)^. Cohort studies further demonstrated that a particular subset of the CD8^+^ cells, the CD38^+^ cells, were elevated in individuals who developed AIDS rapidly and suggested that “activation” of immune cells plays a key role in progression of HIV disease^9^^)^. A cohort study demonstrated that HIV-infected individuals from whom cytomegalovirus (CMV) was frequently isolated from the semen had a more rapid progression to AIDS, supporting the hypothesis that activation of immune cells, which occurs with infection by viruses such as CMV, plays a role in progression of HIV disease^10^^)^. Other studies have demonstrated that cytotoxic CD8^+^ cells are the major line of defense against HIV infection^11^^)^. The importance of viral load in progression of HIV disease is demonstrated in Figure 5, taken from a cohort study which demonstrates the importance of viral load early in infection in predicting the subsequent rate of progression to clinical AIDS at different levels of immune capacity represented by the different levels of CD4^+^ cells^12^^)^. These studies have demonstrated the interplay between the virus and the immune response in the production of HIV disease. Thus they have been very useful for deciding when and how to treat HIV-infected individuals to maximize therapeutic efficacy.

Epidemiologic studies have also demonstrated the importance of genetic factors in the natural history of HIV infection. A nested case-control study demonstrated that individuals who lack the gene for the CCR-5 receptor site on a particular immune cell, the CD4^+^ cell, are resistant to infection by HIV because the virus is unable to attach itself to the cell^13^^)^. These studies have suggested that blocking of CD4^+^ receptors through drugs may be a successful strategy for preventing infection of CD4^+^ cells, thus slowing or preventing progression of HIV disease. Other nested case-control studies have suggested that individuals who are resistant to HIV have a different distribution of TAP variants and HLA alleles than individuals who are susceptible^5^^)^. These studies have suggested that antigen processing and presentation are key factors which determine the ability of the host to mount an effective immune response. On the basis of this information a new group of preventive and therapeutic strategies/drugs are being developed.

## EVALUATION OF THERAPEUTIC AND INTERVENTION STRATEGIES

Clinical trials can demonstrate the biologic efficacy of therapy, but in the real world therapy is not usually administered under the stringent conditions possible in clinical trials. The effectiveness of therapy as used in the real world can be evaluated using epidemiologic strategies. In the example given in Table 3 an innovative cohort study design was used to demonstrate the impact of highly active antiretroviral therapy (HAART) on HIV-infected men not in a clinical trial^14^^)^. Therapeutic regimens were characterized by using calendar periods in which they were the dominant mode of treatment given to the men in the MACS study. Thus, in the period 1990-1993, monotherapy, usually with zidovudine, was the only regimen given. In the period 1993-June 1995, combination therapy was the major regimen given, and in the period July 1995-June 1997, HAART became the major regimen for treatment. From Table 3 it is clear that the use of combination therapy was not associated with increased time to AIDS and survival, but that there was a significant increase in both with the use of HAART. Thus HAART has not only biologic efficacy but also public health effectiveness as a therapeutic strategy.

**Table 3.** Relative AIDS-free and survival times.

	Times to AIDS	Times to Death
Calendar		
1999.0 to 1930.0	1	1

1993.0 to 1995.5	0.97	1.01
(95% C.I.)	(0.86, 1.09)	(0.91, 1.12)

1995.5 to 1997.5	1.63	1.21
(95% C.I.)	(1.40, 1.89)	(1.07, 1.36)

**Figure 6.**
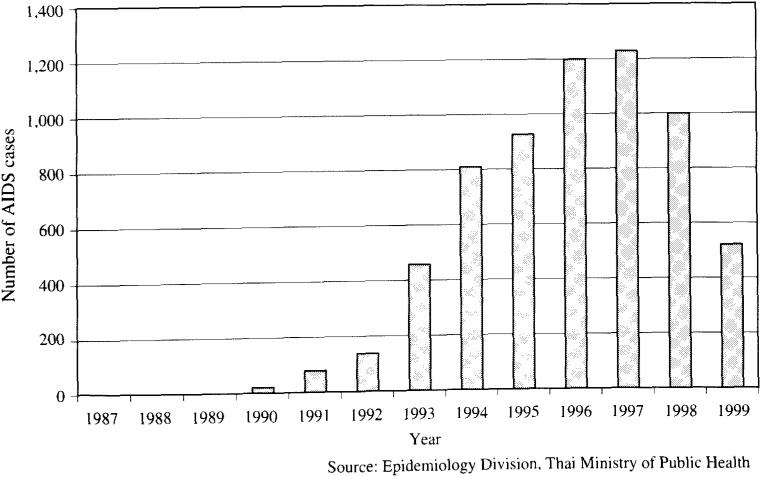
Annual number of AIDS cases in children in Thailand, 1987-1999.

One of the most tragic aspects of the HIV pandemic is the infection of children from their HIV-infected mothers during delivery. In 1995-1996 clinical trials conducted in Thailand demonstrated that the proportion of children infected with HIV could be reduced from 19% to 8% by administering zidovudine to the mother for two weeks prior to and during delivery^15^^)^. This study had immediate policy implications; in 1997 the Thai government implemented an intervention policy of treatment of infected mothers with zidovudine. Surveillance for reported cases of HIV-infected infants for the years before and after the implementation of the policy of treating HIV-infected pregnant women is shown in Figure 6. The impact of the policy is clearly seen in the dramatic reduction in the number of reported HIV-infected infants following implementation of the policy.

## VOLUNTEERS

Much of the credit for the success of epidemiologic studies of HIV/AIDS goes to the men and women who have volunteered to participate in these studies. Often they had to do so at the risk of stigmatization resulting from disclosure of their sexual orientation and/or their HIV/AIDS status. They were willing to take these risks in the hope of contributing to the prevention and ultimate control of HIV infection and AIDS. Epidemiologists and the world should be grateful to these selfless people without whom these studies would not have been possible.

Innovative epidemiologists will continue to apply epidemiologic principles and strategies to resolution of the HIV/AIDS epidemic as well as future, as yet unknown, challenges to the health of the public.
